# Arm rehabilitation in post stroke subjects: A randomized controlled trial on the efficacy of myoelectrically driven FES applied in a task-oriented approach

**DOI:** 10.1371/journal.pone.0188642

**Published:** 2017-12-04

**Authors:** Johanna Jonsdottir, Rune Thorsen, Irene Aprile, Silvia Galeri, Giovanna Spannocchi, Ettore Beghi, Elisa Bianchi, Angelo Montesano, Maurizio Ferrarin

**Affiliations:** 1 IRCCS Don Gnocchi Foundation Onlus, Milan, Italy; 2 IRCCS Istituto di Ricerche Farmacologiche Mario Negri, Milan, Italy; IRCCS E. Medea, ITALY

## Abstract

**Purpose:**

Motor recovery of persons after stroke may be enhanced by a novel approach where residual muscle activity is facilitated by patient-controlled electrical muscle activation. Myoelectric activity from hemiparetic muscles is then used for continuous control of functional electrical stimulation (MeCFES) of same or synergic muscles to promote restoration of movements during task-oriented therapy (TOT). Use of MeCFES during TOT may help to obtain a larger functional and neurological recovery than otherwise possible.

**Study design:**

Multicenter randomized controlled trial.

**Methods:**

Eighty two acute and chronic stroke victims were recruited through the collaborating facilities and after signing an informed consent were randomized to receive either the experimental (MeCFES assisted TOT (M-TOT) or conventional rehabilitation care including TOT (C-TOT). Both groups received 45 minutes of rehabilitation over 25 sessions. Outcomes were Action Research Arm Test (ARAT), Upper Extremity Fugl-Meyer Assessment (FMA-UE) scores and Disability of the Arm Shoulder and Hand questionnaire.

**Results:**

Sixty eight subjects completed the protocol (Mean age 66.2, range 36.5–88.7, onset months 12.7, range 0.8–19.1) of which 45 were seen at follow up 5 weeks later. There were significant improvements in both groups on ARAT (median improvement: MeCFES TOT group 3.0; C-TOT group 2.0) and FMA-UE (median improvement: M-TOT 4.5; C-TOT 3.5). Considering subacute subjects (time since stroke < 6 months), there was a trend for a larger proportion of improved patients in the M-TOT group following rehabilitation (57.9%) than in the C-TOT group (33.2%) (difference in proportion improved 24.7%; 95% CI -4.0; 48.6), though the study did not meet the planned sample size.

**Conclusion:**

This is the first large multicentre RCT to compare MeCFES assisted TOT with conventional care TOT for the upper extremity. No adverse events or negative outcomes were encountered, thus we conclude that MeCFES can be a safe adjunct to rehabilitation that could promote recovery of upper limb function in persons after stroke, particularly when applied in the subacute phase.

## Introduction

Stroke is the leading cause of disability in adults in the world and can result in highly complex clinical situations. The insult often involves the sensory-motor system leading to hemiparesis and impairment of the upper limb in over 50% of survivors [1,2]. Although some structural recovery is possible, especially in the first months after stroke, only a small percentage of persons recover pre-morbid movement patterns and functionality [3].

Limitations in reaching and grasping have an important role in determining the level of independence of the person in their daily activities and the subsequent impact on their quality of life. Tailored goal oriented rehabilitation is therefore an essential factor in reducing impairment and augmenting functionality of a hemiplegic arm. A plurality of interventions may help the subject to restore participation and adapt to the new clinical status including task oriented therapy (TOT) that has been shown to be effective for motor recovery [4,5], as well as constraint induced movement therapy (CIMT) [6], biofeedback and robot assisted therapy [7–9]. Moreover, electrostimulation has been applied to improve muscle recruitment and aid motor recovery. Since resources and time in rehabilitation are limited it is important to identify and employ effective interventions [10].

The inability to use the arm in an efficient way may lead to non use of the arm and hand that can lead to changes also at the neural level [11]. It is therefore essential that arm use is facilitated in meaningful activities. Approaches that assist the person during purposeful voluntarily activated movement could be important for inducing neuroplasticity and increasing function. Neuromuscular electrical stimulation (NMES) has been employed in rehabilitation of stroke patients either to generate muscle contraction or be a support during movements; however, with inconsistent results [11–20]. A prerequisite for neuroplasticity through training is the volitional intent and attention of the person and it therefore follows that the user should participate consciously in the rehabilitative intervention [21,22].

Through the use of EMG it is technically possible to register the myoelectric activity from voluntary contraction of a muscle while its motor nerve is being stimulated by electrical impulses [23]. MeCFES is a method where the FES is directly controlled by volitional EMG activity. In contrast to EMG triggered FES, the controlling muscle is continuously controlling the stimulation intensity. Thus the resulting movement and intrinsic multisensory activation is synchronized with the active attention and intention of the subject and the muscle contraction can be gradually modulated by the subject himself facilitating motor learning and recovery of function. This has been demonstrated to be possible in spinal cord injured subjects [24,25] and a pilot study has shown that when the controlling and stimulated muscles are homologous or they are synergistic it may lead to a marked increase in motor function of the hemiparetic forearm of selected stroke patients [26]. Motor learning principles required for CNS-activity-dependent plasticity, in fact, include task-oriented movements, muscle activation driving practice of movement, focused attention, repetition of desired movements, and training specificity [21,22,27]. The use of MeCFES during active challenging goal oriented movements should help the patient and the therapist overcome the effect of learned non use by turning attempts to move the arm into successful movements.

We hypothesize that applying MeCFES in a task oriented paradigm to assist normal arm movements during rehabilitation of the upper limb in persons with stroke will improve the movement quality and success and thus induce recovery at the body functions level (impairment) and the activity level (disability) of the International Classification of Function, Disability and Health (ICF) [28] superior to that induced by usual care task-oriented rehabilitation.

## Materials and methods

### Study design

An observer-blinded block-randomized controlled multicenter trial with post-acute stroke patients was carried out. The trial involved four rehabilitation centers of the Don Carlo Gnocchi Foundation located in Milano (two centers, Santa Maria Nascente (SMN), the trial leading center, and Palazzolo), Roma and Rovato. Participants that met the inclusion criteria were allocated to one of two groups; an experimental group (M-TOT) or the control group (C-TOT). In the experimental group the MeCFES was applied to support impaired movements while the participant was working on task-oriented activities under guidance of the therapist. In the control group, participants were treated with standard rehabilitation care that included task-oriented activity. The study was approved by the local ethics committees in April 2010 ("Ethical Committee of IRCCS Don Carlo Gnocchi Foundation"), the first participant was enrolled in April 2011 and the last in August 2015. The study was retrospectively registered in the clinical trials list as ClinicalTrials.gov (NCT03019744). Registration was done retrospectively since at the moment of study beginning year 2011 registrations of clinical trials, although recommended by World Health Organization, were not general practice in our institute. However, we confirm that all ongoing and related trials for this intervention are registered.

### Participants

Adult persons with a first ischemic or haemorrhagic stroke were recruited from collaborating rehabilitation centers within the four-year project. To be included the subjects had to be at least a month post stroke at first evaluation (T0), be able to cognitively, physically and logistically participate in the study, have a minimum voluntary muscle activation of shoulder flexors (at least 1 on the Manual Muscle test (Medical Research Council scale) [29], have a passive range of motion of the shoulder and elbow of more than 90° and no severe spasticity (≤3 Ashworth scale) [30] of upper limb muscles. Exclusion criteria were presence of implanted electronic devices, epilepsy, respiratory insufficiency, pregnancy, peripheral neuropathies, cutaneous ulcers at the stimulation zone and other use of FES on the upper limb.

After signing an informed consent participants were randomized according to four random allocation sequences, generated before the beginning of the study according to time since stroke (subacute ≤ 6 months post stroke or chronic, >6 months post stroke) and functional level (high, FMA-UE score >22 or low FMA-UE≤22). A random order block of 2 (experimental) + 2 (control) assignments was used for each of the four categories. The allocation sequences were concealed from clinicians enrolling patients.

### Evaluation

Outcome measures of the study were the improved 15 item Action Research Arm Test (ARAT) [31], and the Upper Extremity section of the Fugl Meyer scale (FMA-UE) [32], representing respectively the activity level of the ICF and the neurological state at the body function level of the ICF. The Quick version of the Disability of the Arm Shoulder and Hand questionnaire (DASH, score of maximum limitation 100) [33] was used to measure the impact of treatment on participation. The 15 item ARAT is a shorter version of the original ARAT that was suggested after a Rasch analysis of the ARAT carried out by Van der Lee [31] that showed that 4 of the original 19 items were redundant. The improved ARAT assesses the ability to grasp and move objects (maximum total score 45) such as the original version while the FMA is a stroke-specific, performance-based impairment index designed to assess motor functioning (FMA-UE, maximum total score 66). For both ARAT and FMA-UE a higher score indicates better arm function while a higher score on the DASH indicates a greater limitation. A change on the ARAT equal or more than five points is considered an improvement in function. Participants also filled out a VAS for perceived pain (maximum pain score = 10).

Assessments were made by a trained physical therapist, blind to group assignment, at 4 similarly spaced time intervals over 6 weeks, T0, T1, T2 and T3, and T4 at Follow up 5 weeks later. The whole evaluation protocol was administered at baseline (T0), at post-treatment (T3 at week 6) and at 5-week follow-up (T4), while only ARAT was administered at intermediate time points, respectively after 8 (T1) and 16 (T2) rehabilitation sessions.

Eight different physiotherapists served as assessors, two per site. All assessors took part in a training session prior to the commencement of the study. The same assessor performed the serial assessments for each individual participant. Participants were not blinded to the intervention, however they remained naive as to the supposed efficacy of the 2 intervention conditions.

### Intervention

Each subject was assessed and treated in the same rehabilitation center where he/she was enrolled. Treatment and evaluation protocols were agreed upon and therapists in all four centers were specifically trained to use the MeCFES approach in order to reduce variation in treatment protocol application. A number of possible preset protocols had been prepared in collaboration with therapists at the leading site to form an Investigators Brochure with detailed descriptions. The treating therapists underwent a training course and were instructed to read a user manual describing how to apply the MeCFES and giving indications of appropriate TOT exercises with which the MeCFES could be used. Each center thus had a MeCFES device at its disposal and physical therapists were trained to use it.

The intervention protocol for both groups consisted of 25 sessions, lasting 45 minutes each, that were applied 5 days per week over 5–6 weeks. In addition to the 25 daily sessions of the study protocol, all participants received usual care physical therapy as planned for their individual problems.

#### MeCFES added to Task-oriented arm rehabilitation (M-TOT)

The physical therapist used the MeCFES to assist volitional movements of the patients during task-oriented therapy. Based upon the clinical needs of the patient, activity of one or more arm muscles was identified in order to control stimulation of that same muscle or synergic muscles.

One of the commonly used modalities was for example to let wrist extension and anterior deltoid control in synergy with the opening of the hand for reaching. Only when the patient actively used both muscles the stimulation would induce hand opening. A variant of this was to let activity of the long flexors of the fingers inhibit stimulation controlled by wrist extensors, in order to promote unlearning of undesirable co-contraction of antagonist muscles. Therapists were furthermore encouraged to use the last half of the session to let the patient repeat the TOT exercises without the MeCFES in order to promote a carry-over or learning effect. The TOT exercises included movements of reaching, grasping, manipulating and moving appropriate objects.

#### Task-oriented arm rehabilitation (C-TOT)

Usual care arm therapy typically consisted of task-oriented exercises, similar to those in the experimental group, aimed to improve arm functionality.

### Material

The MeCFES device, described in detail elsewhere [23,24], is composed of four independent EMG amplifiers and four outputs for muscle-nerve stimulation. Digital signal processing algorithms reduce stimulation artifacts and transform each input into an estimate of the volitional myoelectric activity of the controlling muscle. These estimates are combined by addition or subtraction to control each of the four FES output generators. The relation between myoelectric effort and stimulation is controlled by a piecewise linear function where offset, gain and a maximum can be adjusted by the therapist using a portable computer. The computer is connected wireless to the MeCFES while the therapist is adjusting these settings. For each stimulation channel, the therapist may also adjust the maximum stimulation intensity as to protect the subject against excessive stimulation. Through a graphical interface the therapist can combine the inputs with outputs and the sign of the gain determines if the input acts as an excitatory or inhibitory component of the correspondent stimulation channel.

### Sample size estimation

A minimal clinically important difference (MCID) of 5 points or more on the improved ARAT between baseline (T0) and post treatment (T3) was considered to divide the sample into improved and not improved [34]. Based on a statistical power of 80% with a two-sided level of significance of 5% and assuming that 75% of patients in the experimental group and 50% in the control group would exceed the MCID (25% in favor of the experimental group), a sample size of 110 subjects was required. A sample size of 120 subjects (sixty per group) was planned to account for an expected dropout of 10%.

### Data collection and statistical analysis

Outcome measures were retrieved on site by the assessing therapist and sent as excel file or fax to the principal investigator and inserted into the trial registry database.

Descriptive statistics for the two treatment arms are reported as means and ranges, medians with interquartile ranges, or counts and percentages. A primary endpoint was defined as an improvement on the ARAT score of 5 points (MCID) or more, from the pre-treatment (T0) to the post-treatment visit (T3): patients were accordingly categorized as improved or not improved. Results are reported as differences in proportions with 95% confidence intervals. The same results are reported for subgroups defined according to stroke chronicity (chronic or subacute).

Secondary endpoints were the changes of ARAT, FMA-UE and DASH scores over the treatment period (T0-T3), and during the follow-up period (T3-T4). These endpoints were evaluated, separately, using different multivariable repeated measures mixed models (with an unstructured variance-covariance matrix). Three different models, with ARAT, FMA-UE, and DASH scores as dependent variables, with treatment group as independent variable, and with stroke chronicity and rehabilitation center as covariates, were used both to evaluate changes during the treatment period (from T0 to T3), and then separately to evaluate changes over the entire follow-up (from T0 to T4). Medians of the differences from baseline scores were reported for each outcome measure at each available visit. Results of the repeated measures models are reported as treatment effect, visit effect and treatment*visit interaction effect. The significance level was set at 5% and all tests were two-tailed. Analyses were performed using SAS 9.2 (SAS Institute Inc., Cary, NC, USA).

## Results

Within the duration of the project (April 2011 and August 2015), 82 patients (mean age years: 66.6, range: 36.5–88.7, mean onset months: 12.6, range: 0.8–190,1) from the four rehabilitation centers were recruited and randomized (M-TOT group, 38; C-TOT group, 44). The last evaluation was performed in October 2015. Fourteen patients left the study before the post-treatment visit (T3): 5 were randomized but dropped out before their baseline assessment (T0) (1 died, 1 had an epileptic seizure, 1 refused the treatment, 2 for unknown reasons); Nine of the 77 patients that had a baseline evaluation (T0) did not finish at least 90% of the rehabilitation sessions and so were considered drop outs resulting in a drop-out rate of almost 12% after baseline evaluation (5 dismissed from center prior to the study end, 1 as being non-compliant with the treatment, 3 for unknown reasons). None of the dropouts was due to dissatisfaction with type of treatment received. Dismissal from the center prior to study end happened for practical reasons, such as the patients being moved to rehabilitation centers nearer to their home. The patient that was non-compliant with the treatment missed many rehabilitation sessions due to sickness and so was unable to finish the protocol in time.

**Fig 1:**
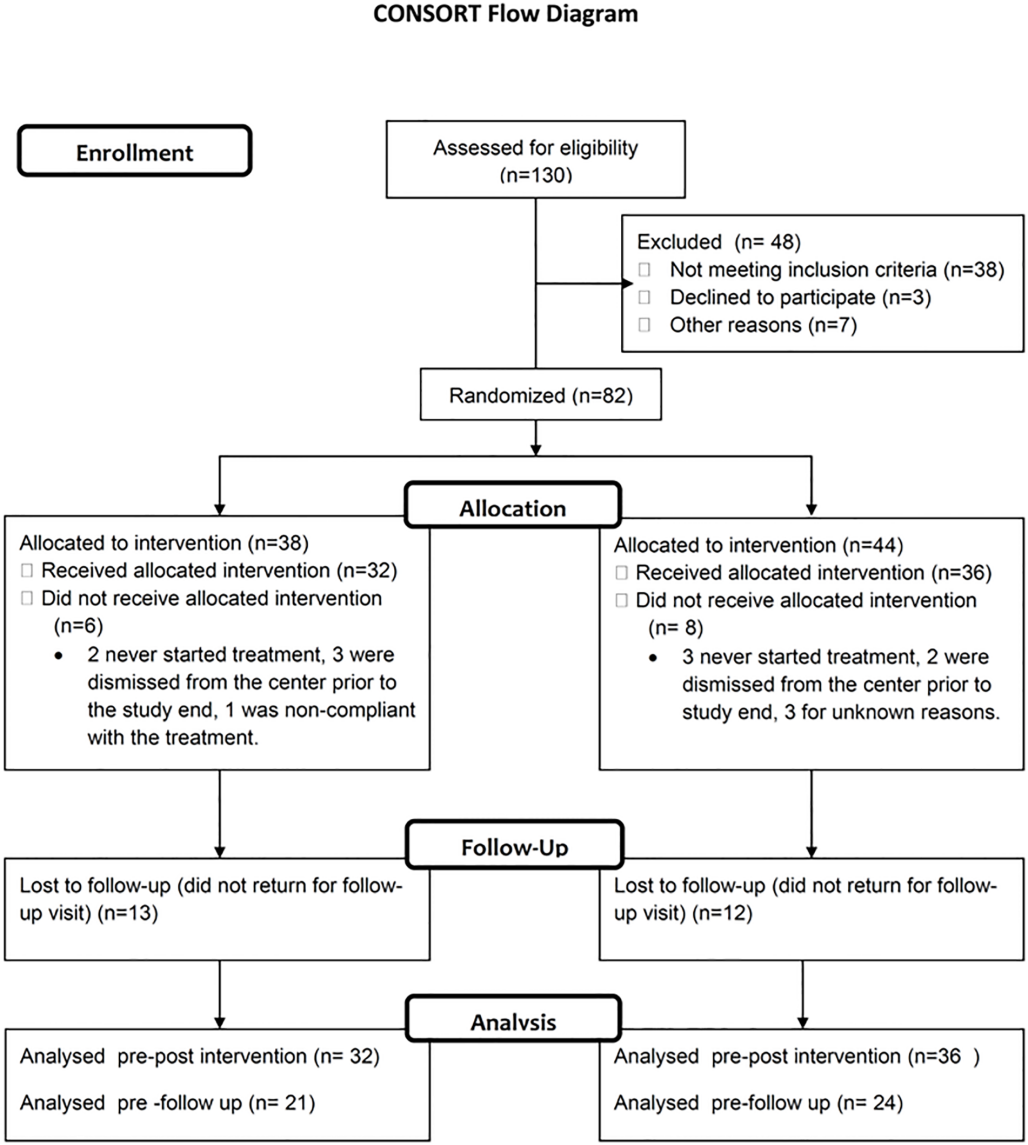
CONSORT flow-chart.

Sixty eight patients (experimental group, 32; control group, 36) completed the assigned treatment and 45 could be seen at the 5-week follow-up visit (T4) (see CONSORT flow-chart in Fig 1). The low rate of patients turning up for a follow up is of concern, but in all cases it was because it was inconvenient for the persons, they lived too far away from the center or did not have anyone that could assist them in getting to the center.

No adverse events or negative outcomes were encountered during the treatment period. The median VAS for pain was 0 at baseline and remained unchanged which implies that both treatments were safe and did not lead to an increase in shoulder pain.

The demographic and clinical features of the 68 patients with post-treatment evaluation are illustrated in Table 1. The mean age at the baseline visit was 65.9 years for the M-TOTgroup and 67.9 years for the C-TOT group, with a mean time since stroke of 20.1 and 10.1 months, respectively. Females predominated in both groups, and ischemic stroke was the most common type. Subacute patients were more represented in the C-TOT group (75%) than in the M-TOT group (59%).

**Table 1:** Demographic and clinical features of experimental and control groups.

Variable	M-TOT (N = 32)			C-TOT (N = 36)		
	N	Median	Range	N	Median	Range
**Age (years)**	32	68.3	36.5–84.8	36	67.7	38.0–88.7
**Disease duration (months)**	32	4.5	0.9–190.1	36	3	0.9–97.4
	**N**	**%**		**N**	**%**	
**Sex**						
F	19	59.4		20	55.6	
M	13	40.6		16	44.4	
**Stroke type**						
Hemorrhagic	5	15.6		7	19.4	
Ischemic	26	81.3		29	80.6	
Ischemic and hemorrhagic	1	3.1		0	0.0	
**Stroke site**						
Cortical	5	15.6		8	23.5	
Cortical and subcortical	11	34.4		15	44.1	
Subcortical	11	34.4		9	26.5	
Brainstem	5	15.6		2	5.9	
Not specified				2		
**Chronicity**						
Chronic	13	40.6		9	25.0	
Subacute	19	59.4		27	75.0	
**Rehabilitation center**						
Milano (Palazzolo)	2	6.2		7	19.4	
Roma	11	34.4		11	30.6	
Rovato	6	18.8		7	19.4	
Milano (SMN)	13	40.6		11	30.6	

N: number; M-TOT: Myoelectric control of functional electrical stimulation-task oriented training; C-TOT: control-task oriented training.

All analyses of treatment effect subsequently reported were performed in the 68 subjects with post-treatment evaluation (See Table 2 for results of all outcome variables expressed as medians, means and range). Median baseline score of ARAT was 6 and 6.5 out of a total score of 45 in the M-TOT and C-TOT group respectively while following treatment the median scores were 21 and 12.5 respectively. Median FMA-UE score at baseline was 28 and 32 out of a total score of 66 respectively for the M-TOT and C-TOT group and following treatment it was 39 and 36, respectively.

**Table 2:** Outcome assessments at baseline and post intervention.

Test		M-TOT (N = 32)			C-TOT (N = 36)		
		Median	Range	Mean(SD)	Median	Range	Mean (SD)
**ARAT**	PRE	6.0	0–43	13.9 (16.1)	6.5	0–44	15.1 (16.4)
	POST	21.0	0–45	20.5 (17.1)	12.5	0–45	20.0 (18.7)
**FMA-UE**	PRE	28.0	4–64	29.4 (19.4)	32.0	2–63	30.4 (19.9)
	POST	39.0	8–66	37.0 (19.9)	36.0	8–66	34.9 (21.9)
**DASH**	PRE	62.5	8–68	60.8 (17.5)	54.5	14–75	51.0 (18.3)
	POST	56.5	18–71	55.9 (17.9)	52.5	6–66	44.2 (20.6)

IQR: interquartile range; SD: standard deviation; M-TOT: Myoelectric control of functional electrical stimulation-task oriented training; C-TOT: control-task oriented training. ARAT: action research arm test; FMA-UE: Fugl Meyer assessment for the upper limbs; DASH: disability of the arm, shoulder and hand questionnaire.

**Fig 2:**
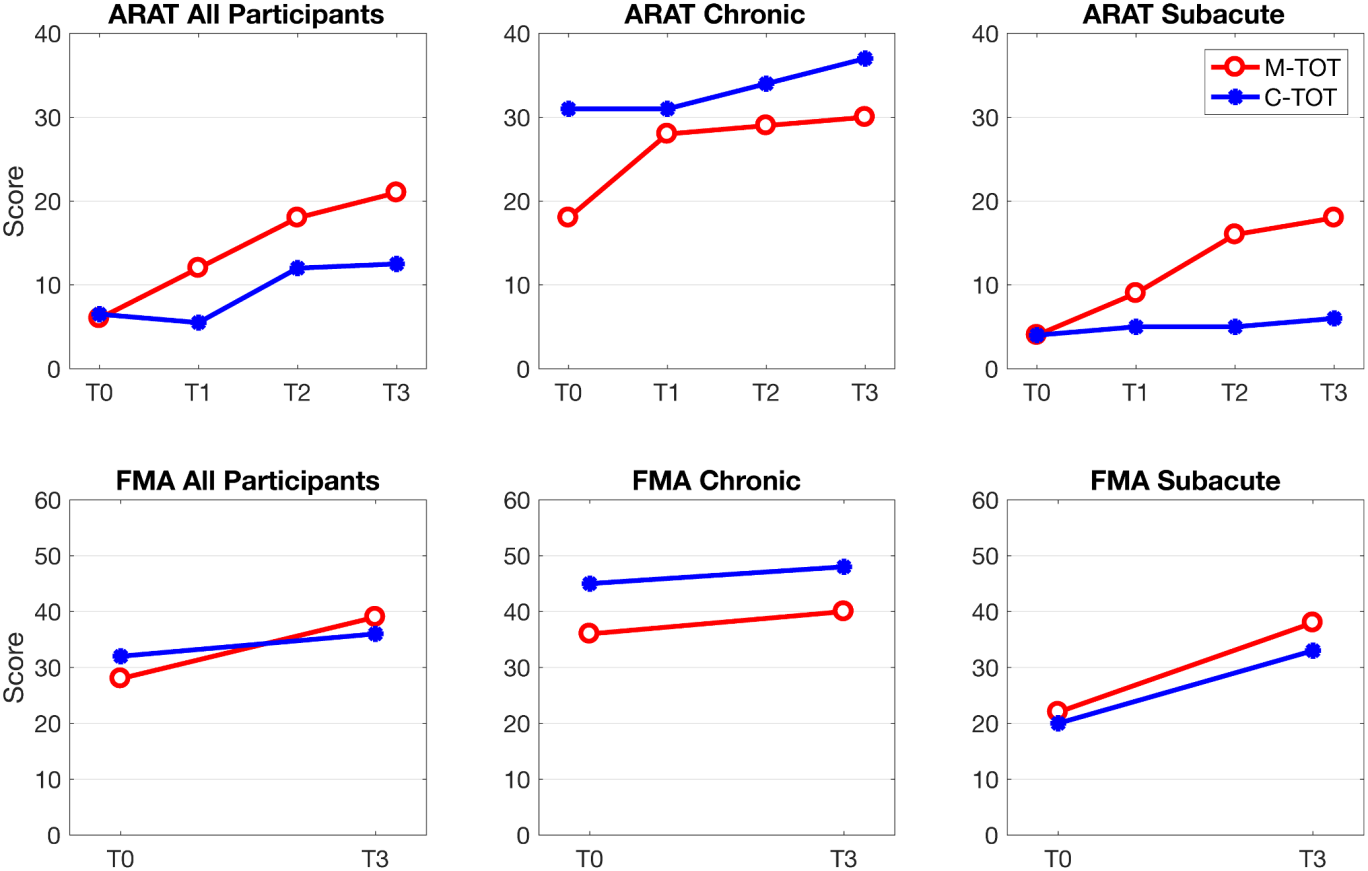
Change over time of median ARAT and FMA-UE scores for M-TOT and C-TOT groups, and their subgroups. ARAT: Action research arm test; FMA-UE: Fugl Meyer assessment for the upper limbs. M-TOT: Myoelectric control of functional electrical stimulation-task oriented training; C-TOT: control-task oriented training. T0: baseline visit; T1: after 8 rehabilitation sessions; T2: after 16 rehabilitation sessions; T3: post-treatment visit (after 25 rehabilitation sessions, 6–7 weeks).

Fig 2 depicts the change over time of the median ARAT and FMA-UE scores for both groups and subgroups, chronic and subacute, at baseline and post intervention.

The results of the primary endpoint analysis are shown in Table 3. In the M-TOT group, 14 patients out of 32 (43.8%) showed an improvement of 5 points or more on the ARAT score at the post-treatment assessment. The number of improved patients in the C-TOT group was 12 out of 36 (33.3%). No significant differences were observed between the two treatment arms (difference in proportions: 10.5; 95% CI: -12.2; 31.8). Subgroup analyses showed that “usual care" TOT had a similar effect in chronic (33.3% improved) and subacute patients (33.2% improved), while M-TOT appeared to be more effective in subacute (57.9% improved) than in chronic patients (23.1% improved); nevertheless, neither in chronic (difference in proportions: -10.2; 95% CI: -44.9; 24.3) nor in subacute (difference in proportions: 24.7; 95% CI: -4.0; 48.6) subgroups, was a significant difference found between the two treatment arms. When adjusting for stroke chronicity and centers results remained substantially unchanged (data not shown).

**Table 3:** Number of improved patients (ΔARAT_pre-post_ ≥5) at post-treatment visit in the M-TOT and the C-TOT group, with differences and confidence intervals.

	M-TOT (N = 32)	C-TOT (N = 36)	p-value	Difference in proportions	95% CI
	N Improved (%)	N improved (%)		%	
All patients	14 (43.8)	12 (33.3)	0.45	10.5	-12.2; 31.8
Chronic stroke	3 (23.1)	3 (33.3)	0.65	-10.2	-44.9; 24.3
Subacute stroke	11 (57.9)	9 (33.2)	0.09	24.7	-4.0; 48.6

CI: confidence interval; M-TOT: Myoelectric control of functional electrical stimulation-task oriented training; C-TOT: control-task oriented training; N improved: number improved. ARAT: action research arm test.

Medians of the changes from baseline of ARAT, FMA-UEand DASH scores at each available treatment evaluation are shown in Table 4 along with the results of the repeated measures analysis. There was a significant improvement from baseline to post-treatment scores for all outcome measures in both groups; however, no significant interactions between treatment and visit were detected (Table 3), meaning that the change from baseline was similar in the two treatment groups, although FMA-UE showed a trend for interaction (p = 0.07) with bigger changes in the M-TOTgroup. The median change scores for ARAT from baseline to the end of treatment: were 3.0 in the MeCFES group, and 2.0 in the C-TOT group; for FMA_UE 4.5 in the M-TOT group, and 3.5 in the C-TOT group; and for DASH -4.5 in the M-TOT group, and -4.8 in the C-TOT group.

**Table 4:** Median differences of ARAT, FMA-UE and DASH scores from baseline (T0) to post-treatment visit (T3).

Test	Time	M-TOT (N = 32)			C-TOT (N = 36)			p-value (treatment)	p-value (visit)	p-value (treatment*visit)
		N	Median	IQR	N	Median	IQR			
**ARAT**	**T1**	31	0.0	0.0;5.0	34	0.0	0.0;2.0			
	**T2**	32	2.0	0.0;11.0	33	1.0	0.0;7.0			
	**T3**	32	3.0	0.0;12.5	36	2.0	0.0;9.5	0.77	<0.0001	0.27
**FMA-UE**	**T3**	32	4.5	1.5;14.0	36	3.5	0.5;6.5	0.58	<0.0001	0.07
**DASH**	**T3**	32	-4.5	-11.4;1.5	34	-4.8	-14.0;0	0.07	<0.0001	0.32

M-TOT: Myoelectric control of functional electrical stimulation-task oriented training; C-TOT: control-task oriented training; IQR: interquartile range. ARAT: action research arm test; FMA-UE: Fugl Meyer assessment for the upper limbs; DASH: disability of the arm, shoulder and hand questionnaire.T0: baseline visit; T1: after 8 rehabilitation sessions; T2: after 16 rehabilitation sessions; T3: post-treatment visit (after 25 rehabilitation sessions, 6–7 weeks). p-values for treatment effect (differences between treatment arms), visit effect (differences between visits) and treatment*visit interaction (differences in the change over time between treatment arms) were obtained from a multivariable repeated measures mixed model, adjusting by stroke chronicity and rehabilitation center.

### Follow up measures

The same outcome measures were analyzed in the 45 patients with the 6-week follow-up assessment (Table 5). Repeated measures models, with an additional time point for the follow-up assessment, showed comparable results. In addition, contrasts between T0 and T3, and between T3 and T4 revealed that there was a significant improvement of ARAT, FMA-UE and DASH scores from pre- to post-treatment assessment, and with no subsequent variations until the 6-week follow-up assessment, although there was a trend for continued improvement (p = 0.07) on the FMA-UE from the post-treatment assessment to the follow up.

**Table 5:** Median differences of ARAT, FMA-UEand DASH scores from baseline (T0) to Post (T3) and to the 6-week follow-up visit (T4).

Test	Time	M-TOT (N = 21)			C-TOT (N = 24)			p-value (treatment)	p-value (visit)	p-value (treatment*visit)	p-value (visit contrasts)
		N	Median	IQR	N	Median	IQR				
**ARAT**	**T1**	20	1.0	0.0;6.5	23	0.0	0.0;2.0	0.78	0.0006	0.13	
	**T2**	21	2.0	0.0;10.0	21	0.0	0.0;4.0				
	**T3**	21	4.0	0.0;14.0	24	1.5	0.0;5.0				T0-T3: <0.0001
	**T4**	21	4.0	0.0;14.0	24	3.0	0.0;4.0				T3-T4: 0.62
**FMA-UE**	**T3**	21	5.0	2.0;14.0	24	3.5	0.5;6.5	0.37	<0.0001	0.13	T0-T3: <0.0001
	**T4**	21	7.0	2.0;15.0	24	4.0	0.0;7.0				T3-T4: 0.07
**DASH**	**T3**	21	-6.8	-13.6;-2.3	22	-4.5	-13.6;0	0.10	<0.0001	0.81	T0-T3: <0.0001
	**T4**	21	-10.5	-18.2;-2.3	19	-16.5	-23.0;0				T3-T4: 0.31

M-TOT: Myoelectric control of functional electrical stimulation-task oriented training; C-TOT: control-task oriented training; IQR: interquartile range. ARAT: action research arm test; FMA-UE: Fugl Meyer assessment for the upper limbs; DASH: disability of the arm, shoulder and hand questionnaire.T1: after 8 rehabilitation sessions; T2: after 16 rehabilitation sessions; T3: post-treatment visit (after 25 rehabilitation sessions, 6–7 weeks).; T4: 6-week follow-up visit. p-values for treatment effect (differences between treatment arms), visit effect (differences between visits), treatment*visit interaction (differences in the change over time between treatment arms) and visit contrasts (change between selected visit times) were obtained from a multivariable repeated measures mixed model, adjusting by stroke chronicity and rehabilitation center.

## Discussion

This multicenter, randomized controlled trial investigated the effect of adjunct of myoelectrically controlled functional electrostimulation (MeCFES) to task oriented training (TOT) of the affected arm in persons with stroke as compared to a control group that received the same amount of TOT without electrical stimulation. The MeCFES applied to forearm extensors during task oriented activity led to improved arm function at the neurological and activity level similar to that observed for usual care TOT of the arm, however, there were some indications that adding MeCFES was of more benefit to the persons in the subacute phase after stroke than a usual TOT. The planned sample size was not reached though and so definite conclusions as to a greater effectiveness of MeCFES-assisted TOT than usual TOT cannot be made. However, the results represent an important achievement that prompt further investigation.

These findings are in accordance with reviews of the literature done on the influence of NMES on arm motor recovery in persons after stroke. The general conclusion is that, while there are indications of benefit for arm function, strong evidence is still missing, especially when compared to other valid therapies such as TOT, used in the present study [11,12,14]. The positive response of both groups in the present study to task-oriented intervention is in line with findings of some authors that have included TOT [4,35,36] but in contrast with others [37]. These differences in findings may partly be due to differences in chronicity of stroke and differences in task-oriented activities proposed in the studies.

While both the M-TOT group and the C-TOT group improved their arm function, the proportion of clinically meaningfully improved subjects, denoted as an improvement of 5 points or more on the ARAT, was more than 10% higher in the M-TOT group (44% improved) than in the control group (33% improved). Further, while at baseline both groups with a median total ARAT score of 6 (M-TOT) and 6,5 (C-TOT) would be described as having no arm-hand capacity as classified by Nijland [38], at the post assessment the total score was 21 in the M-TOT group indicating that from being a group with no arm-hand capacity they had arrived at the borderline of being a limited arm-hand capacity group. The C-TOT group, with a final score of 13, would instead be classified as having poor arm-hand capacity. There was also a significant reduction in arm deficit in both groups as denoted by the FM-UE with a change in group median score from 28 points to 39 points in the M-TOT group while the C-TOT group had a change from 32 to 36 points. There was thus a trend for bigger change in favor of the M-TOT group indicating that adding MeCFES to a TOT protocol may have greater effect at the neuromotor level of arm function than does usual care TOT.

Several factors, besides the sample size necessary not being reached, may have influenced the inconclusive results of the present study. One of them is the amount of task specific practice with the stimulation on in every session. It may not have been enough to improve arm function in a significant manner beyond that of the TOT alone received by the control group [39]. In the present study the treatment time in each session was approximately 45 minutes, but this time included also the setup of the electrodes/stimulation parameters and TOT without stimulation (in the last half of the session), so the effective MeCFES treatment time was only about 20 minutes. Thus it is possible that with longer stimulation time effects would have been bigger similar to the treatment time effect seen in the study by Page and colleagues [39].

Another aspect that can have confounded the results is the functional heterogeneity of the participants. This study was carried out on persons with both subacute and chronic stroke, the persons also varied greatly in functional arm deficits and there was both a floor and ceiling effect on the ARAT. Improvement in arm function following rehabilitation in general appears to be tied strongly to the severity of arm disability [9,20]. In fact, it is known that the prognosis of arm recovery is quite poor for those with severe to complete upper limb paresis, while persons with mild to moderate upper limb paresis have a better chance of recovering with almost all of them achieving some dexterity with time and in response to rehabilitation [3,40–43]. In the present study a number of persons had severe upper limb paresis at the beginning of the study, resulting in a 0 on the ARAT. Since the persons with 0 on the baseline ARAT score were similarly represented in both treatment groups (from 62–69%) it is unlikely that they contributed to differences between groups but it may have influenced the within group results. In the Excite study, a large multicenter study, Kwakkel and colleagues [20] saw no effect at all from NMES treatment of wrist and finger extension in a large group of subacute subjects that did not have at least 10% of voluntary wrist or finger extension against gravity. Cauraugh and colleagues [43] instead saw a positive impact of stimulation in their chronic subjects that improved, but unlike the study of Kwakkel and colleagues, an inclusion criterion was that they had at least 10° of voluntary movement at the wrist at the beginning of the study. It is thus likely that some voluntary extension is required for treatment effect of electrostimulation in both subacute and chronic stroke, with persons with mild to moderate hand impairment being more likely to improve whether they be subacute or chronic [8,42,44,45].

Subacute patients are, however, more likely to have an improvement in response to whichever treatment, due also to concomitant spontaneous recovery [9,41]. In the present study there appeared to be a further beneficial effect of adding electrical stimulation to the treatment protocol for the subacute participants, with approximately 60% (N = 11/19) improving 5 points or more on the ARAT against one-third (N = 9/27) of the subacute participants in the control group. The between group difference of number of persons improved was near significance (p = 0.098) suggesting that the benefit of adding EMG controlled stimulation to a Task-oriented arm rehabilitation might have been proven for persons in the subacute phase after stroke if the planned sample size had been reached. The trend is underlined by a visual analysis of Fig 2 depicting both ARAT and FMA-UE for both groups and subgroups. This trend is in accordance with findings from several studies that have found a benefit trend from the application of EMG triggered neuromuscular electrical stimulation to wrist and finger extension in subacute persons after stroke [44,46,47].

Based on the present findings, in future studies a total of 63 subacute patients should be enrolled in each treatment arm in order to reach an 80% power and a 5% level of significance.

Forty five participants were available for a 5 week follow-up assessment, approximately 2/3 of each group. Both groups retained the benefit of the intervention at follow up and there was a nonsignificant (p = 0.07) trend for further reduction of impairment in the MeCFES group that had gained a median of 7 points on the FM-UE at the 5 weeks follow up while the control group had gained a median of 4 points from baseline. This indicates that there may be a benefit beyond the stimulation period on the neuromotor function.

Self perceived participation and activity improved equally in both groups indicating a benefit from the intensity of the task-oriented approach in the study rather than a specific benefit of adding the MeCFES. Pain levels at the shoulder were low in both groups at baseline and remained so at the end of the study indicating that both approaches were beneficial for increasing activity and perceived health status without inducing an increase in pain.

### Limitations and generalisability

There are several limitations that must be considered for future studies. The predicted sample size was not reached within the project’s time span which led to insufficient power to draw definitive conclusions as to the efficacy of the MeCFES when applied to arm muscles of persons after stroke during TOT exercises. Moreover, this is a multicenter study which made it difficult to standardize the use of the MeCFES during the rehabilitation sessions. Although efforts were made to standardize the intervention among centers by appropriate training, this may have been insufficient. The MeCFES requires understanding of the technological aspects of the device in order to successfully apply it and the optimal electrode configuration has to be found in every session. The actual use of the device may have differed between centers though every attempt was made to standardize the technical assistance to the physical therapist using the device.

Attrition is the last limitation. Fourteen of the 82 patients enrolled in the study failed to provide some follow-up data, of those 9 patients had a baseline assessment. Our measures may have been affected by such missing data. However, the baseline characteristics of dropouts with only the first assessment did not differ from patients who completed the study (data not shown).

The results of this study are applicable to people with a minimal to severe deficit of the upper extremity in the subacute and chronic period after first stroke.

## Conclusion

This is the first large multicentre RCT to compare MeCFES assisted task oriented therapy with usual care that included a task-oriented component for the upper extremity. Both groups improved their arm function, indicating a general benefit from task-oriented arm rehabilitation, although more people improved with the addition of MeCFES. In particular, subjects who were in the subacute period after stroke appeared to benefit from adding the MeCFES to their training protocol. No adverse events or negative outcomes were encountered, thus it can be concluded that MeCFES may be a safe and promising adjunct to rehabilitation that can help promote recovery of upper limb function in persons after stroke.

## Supporting information

S1 Protocol (DOCX)

S1 CONSORT checklist (DOC)

S1 Dataset (XLS)

## References

[1] Strong K, Mathers C, Bonita R. Preventing stroke: saving lives around the world. The Lancet Neurology. 2007; 6(2), 182–187. doi: 10.1016/S1474-4422(07)70031-5 17239805 10.1016/S1474-4422(07)70031-5

[2] Duncan PW, Goldstein LB, Matchar D, Divine GW, Feussner J. Measurement of motor recovery after stroke. Outcome assessment and sample size requirements. Stroke. 1992;23(8), 1084–1089. 1636182 10.1161/01.str.23.8.1084

[3] Kwakkel G, Kollen BJ, van der Grond J, Prevo AJ. Probability of regaining dexterity in the flaccid upper limb: impact of severity of paresis and time since onset in acute stroke. Stroke. 2003;34:2181–6. doi: 10.1161/01.STR.0000087172.16305.CD 12907818 10.1161/01.STR.0000087172.16305.CD

[4] Van Peppen RP, Kwakkel G, Wood-Dauphinee S, Hendriks HJ, Van der Wees PJ, Dekker J. The impact of physical therapy on functional outcomes after stroke: what's the evidence? Clin Rehabil. 2004;18:833–62. doi: 10.1191/0269215504cr843oa 15609840 10.1191/0269215504cr843oa

[5] Bayona NA, Bitensky J, Salter K, Teasell R. The role of task-specific training in rehabilitation therapies. Topics in stroke rehabilitation. 2005 7 1;12(3):58–65. doi: 10.1310/BQM5-6YGB-MVJ5-WVCR 16110428 10.1310/BQM5-6YGB-MVJ5-WVCR

[6] Taub E. The behavior-analytic origins of constraint-induced movement therapy: an example of behavioral neurorehabilitation. The Behavior Analyst. 2012 10 1;35(2):155–78. 23449867 10.1007/BF03392276 PMC3501420

[7] Jonsdottir J, Cattaneo D, Recalcati M, Regola A, Rabuffetti M, Ferrarin M, et al Task-oriented biofeedback to improve gait in individuals with chronic stroke: motor learning approach. Neurorehabil Neural Repair. 2010;24; 478 doi: 10.1177/1545968309355986 20053951 10.1177/1545968309355986

[8] Kwakkel G, Kollen BJ, Krebs HI. Effects of robot-assisted therapy on upper limb recovery after stroke: a systematic review. Neurorehabilitation and neural repair. 2008 3;22(2):111–21. doi: 10.1177/1545968307305457 17876068 10.1177/1545968307305457 PMC2730506

[9] Langhorne P, Coupar F, Pollock A. Motor recovery after stroke: a systematic review. The Lancet Neurology. 2009 8 31;8(8):741–54. doi: 10.1016/S1474-4422(09)70150-4 19608100 10.1016/S1474-4422(09)70150-4

[10] Kwakkel G, van Wegen EE, Meskers CM. Invited commentary on comparison of robotics, functional electrical stimulation, and motor learning methods for treatment of persistent upper extremity dysfunction after stroke: a randomized controlled trial. Archives of physical medicine and rehabilitation. 2015 6 30;96(6):991–3. doi: 10.1016/j.apmr.2015.02.004 25687763 10.1016/j.apmr.2015.02.004

[11] Nudo RJ, Plautz EJ, Frost SB. Role of adaptive plasticity in recovery of function after damage to motor cortex. Muscle & nerve. 2001 8 1;24(8):1000–19. 11439375 10.1002/mus.1104

[12] Pomeroy VM, King L, Pollock A, Baily-Hallam A, Langhorne P. Electrostimulation for promoting recovery of movement or functional ability after stroke. Cochrane Database of Systematic Reviews. 2006(2). 10.1002/14651858.CD003241.pub2 PMC6465149 16625574

[13] de Kroon JR, Ijzerman MJ, Chae J, Lankhorst GJ, Zilvold G. Relation between stimulation characteristics and clinical outcome in studies using electrical stimulation to improve motor control of the upper extremity in stroke. J Rehabil Med. 2005;37(2):65–74. doi: 10.1080/16501970410024190 15788340 10.1080/16501970410024190

[14] Quandt F, Hummel FC. The influence of functional electrical stimulation on hand motor recovery in stroke patients: a review. Experimental & translational stroke medicine. 2014 8 21;6(1):9 doi: 10.1186/2040-7378-6-9 25276333 10.1186/2040-7378-6-9 PMC4178310

[15] Dorsch S, Ada L, Canning CG. EMG-triggered electrical stimulation is a feasible intervention to apply to multiple arm muscles in people early after stroke, but does not improve strength and activity more than usual therapy: a randomized feasibility trial. Clinical rehabilitation. 2014;1;28(5):482–90. doi: 10.1177/0269215513510011 24198342 10.1177/0269215513510011

[16] Thorsen R, Dalla Costa D, Chiaramonte S, Binda L, Beghi E, Redaelli T, et al A noninvasive neuroprosthesis augments hand grasp force in individuals with cervical spinal cord injury: The functional and therapeutic effects. The Scientific World Journal. 2013 12 30;2013. 10.1155/2013/836959 PMC3893005 24489513

[17] Shin HK, Cho SH, Jeon HS, Lee YH, Song JC, Jang SH, et al Cortical effect and functional recovery by the electromyography-triggered neuromuscular stimulation in chronic stroke patients. Neuroscience letters. 2008 9 19;442(3):174–9. doi: 10.1016/j.neulet.2008.07.026 18644424 10.1016/j.neulet.2008.07.026

[18] Alon G, Levitt AF, McCarthy PA. Functional electrical stimulation enhancement of upper extremity functional recovery during stroke rehabilitation: a pilot study. Neurorehabilitation and neural repair. 2007 5;21(3):207–15. doi: 10.1177/1545968306297871 17369518 10.1177/1545968306297871

[19] Thrasher TA, Zivanovic V, McIlroy W, Popovic MR. Rehabilitation of reaching and grasping function in severe hemiplegic patients using functional electrical stimulation therapy. Neurorehabilitation and neural repair. 2008 11;22(6):706–14. doi: 10.1177/1545968308317436 18971385 10.1177/1545968308317436

[20] Kwakkel G, Winters C, Van Wegen EE, Nijland RH, Van Kuijk AA, Visser-Meily A, et al Effects of unilateral upper limb training in two distinct prognostic groups early after stroke: the EXPLICIT-stroke randomized clinical trial. Neurorehabilitation and neural repair. 2016 10;30(9):804–16. doi: 10.1177/1545968315624784 26747128 10.1177/1545968315624784

[21] Nudo RJ, Plautz EJ, Frost SB. Role of adaptive plasticity in recovery of function after damage to motor cortex. Muscle & nerve. 2001 8 1;24(8):1000–19. 11439375 10.1002/mus.1104

[22] Subramanian SK, Massie CL, Malcolm MP, Levin MF. Does provision of extrinsic feedback result in improved motor learning in the upper limb poststroke? A systematic review of the evidence. Neurorehabil Neural Repair. 2010 2 1;24(2):113–24. doi: 10.1177/1545968309349941 19861591 10.1177/1545968309349941

[23] Thorsen R, Ferrarin M. Battery powered neuromuscular stimulator circuit for use during simultaneous recording of myoelectric signals. Medical engineering & physics. 2009 10 31;31(8):1032–7. doi: 10.1016/j.medengphy.2009.06.006 19620017 10.1016/j.medengphy.2009.06.006

[24] Thorsen R, Spadone R, Ferrarin M. A pilot study of myoelectrically controlled FES of upper extremity. IEEE Transactions on Neural Systems and Rehabilitation Engineering. 2001 6;9(2):161–8. doi: 10.1109/7333.928576 11474969 10.1109/7333.928576

[25] Thorsen R, Occhi E, Boccardi S, Ferrarin M. Functional electrical stimulation reinforced tenodesis effect controlled by myoelectric activity from wrist extensors. Journal of rehabilitation research and development. 2006 3 1;43(2):247–56. 16847791 10.1682/jrrd.2005.04.0068

[26] Thorsen R, Cortesi M, Jonsdottir J, Carpinella I, Morelli D, Casiraghi A, et al Myoelectrically driven functional electrical stimulation may increasemotor recovery of upper limb in poststroke subjects: A randomized controlled pilot study. JRRD. 2013;50(6):785–94. doi: 10.1682/JRRD.2012.07.0123 24203541 10.1682/JRRD.2012.07.0123

[27] Patel AT, Duncan PW, Lai SM, Studenski S. The relation between impairments and functional outcomes poststroke. Arch Phys Med Rehabil. 2000;81(10):1357–63. doi: 10.1053/apmr.2000.9397 11030501 10.1053/apmr.2000.9397

[28] World Health Organization. International Classification of Functioning, Disability and Health: ICF. Geneva:WHO; 2001.

[29] Kendall FP, McCreary EK, Kendall HO. Muscles, Testing and Function: Testing and Function. Lippincott Williams and Wilkins; 1983.

[30] Bohannon RW, Smith MB. Interrater reliability of a modified Ashworth scale of muscle spasticity. Phys Ther. 1987;67(2):206–7. . 3809245 10.1093/ptj/67.2.206

[31] Van der Lee JH, Roorda LD, Beckerman H, Lankhorst GJ, Bouter LM. Improving the Action Research Arm test: a unidimensional hierarchical scale. Clin Rehabil. 2002;16(6):646–53. doi: 10.1191/0269215502cr534oa 12392340 10.1191/0269215502cr534oa

[32] Fugl-Meyer AR, Jaasko IL, Olsson S, Steglind S. The post-stroke hemiplegic patient. SCand J Rehab Med. 1975;7(1341). 1135616

[33] Kennedy CA, Beaton DE, Solway S, McConnell S, Bombardier C. Disabilities of the Arm, Shoulder and Hand (DASH) The DASH and QuickDASH Outcome Measure User’s Manual, Third Edition Toronto, Ontario: Institute for Work & Health, 2011.

[34] Van der Lee JH, de Groot V, Beckerman H, Wagenaar RC, Lankhorst GJ, Bouter LM. The intra-and interrater reliability of the action research arm test: a practical test of upper extremity function in patients with stroke. Archives of physical medicine and rehabilitation. 2001;82(1):14–19. doi: 10.1053/apmr.2001.18668 11239280 10.1053/apmr.2001.18668

[35] Lo AC, Guarino PD, Richards LG, Haselkorn JK, Wittenberg GF, Federman DG, et al Robot-assisted therapy for long-term upper-limb impairment after stroke. New England Journal of Medicine. 2010;13;362(19):1772–83. doi: 10.1056/NEJMoa0911341 20400552 10.1056/NEJMoa0911341 PMC5592692

[36] Wolf SL, Winstein CJ, Miller JP, Taub E, Uswatte G, Morris D, et al Effect of constraint-induced movement therapy on upper extremity function 3 to 9 months after stroke: the EXCITE randomized clinical trial. Jama. 2006;1;296(17):2095–104. doi: 10.1001/jama.296.17.2095 17077374 10.1001/jama.296.17.2095

[37] Winstein CJ, Wolf SL; Dromerick AW, Lane CJ, Nelsen MA, Lewthwaite R, et al Effect of a Task-Oriented Rehabilitation Program on Upper Extremity Recovery Following Motor Stroke The ICARE Randomized Clinical Trial. JAMA. 2016;315(6):571–581. doi: 10.1001/jama.2016.0276 26864411 10.1001/jama.2016.0276 PMC4795962

[38] Nijland RH, van Wegen EE, Harmeling-van der Wel BC, Kwakkel G, EPOS Investigators. Presence of finger extension and shoulder abduction within 72 hours after stroke predicts functional recovery early prediction of functional outcome after stroke: the EPOS cohort study. Stroke. 2010;1;41(4):745–50. doi: 10.1161/STROKEAHA.109.572065 20167916 10.1161/STROKEAHA.109.572065

[39] Page SJ, Levin L, Hermann V, Dunning K, Levine P. Longer versus shorter daily durations of electrical stimulation during task-specific practice in moderately impaired stroke. Arch Phys Med Rehabil. 2012;93(2):200–6. doi: 10.1016/j.apmr.2011.09.016 22289227 10.1016/j.apmr.2011.09.016

[40] Langhorne P, Bernhardt J, Kwakkel G. Stroke rehabilitation. The Lancet. 2011;20;377(9778):1693–702. 10.1016/S0140-6736(11)60325-5 21571152

[41] Veerbeek JM, van Wegen E, van Peppen R, van der Wees PJ, Hendriks E, Rietberg M, et al What is the evidence for physical therapy poststroke? A systematic review and meta-analysis. PloS one. 2014;4;9(2):e87987 doi: 10.1371/journal.pone.0087987 24505342 10.1371/journal.pone.0087987 PMC3913786

[42] Houwink A, Rinske H. Nijland RH, Geurts AC, Kwakkel G. Functional Recovery of the Paretic Upper Limb After Stroke: Who Regains Hand Capacity? Archives of Physical Medicine and Rehabilitation. 2013;94:839–44. doi: 10.1016/j.apmr.2012.11.031 23201317 10.1016/j.apmr.2012.11.031

[43] Cauraugh J, Light K, Kim S, Thigpen M, Behrman A. Chronic motor dysfunction after stroke recovering wrist and finger extension by electromyography-triggered neuromuscular stimulation. Stroke. 2000;1;31(6):1360–4. 10835457 10.1161/01.str.31.6.1360

[44] Knutson JS, Gunzler DD, Wilson RD, Chae J. Contralaterally Controlled Functional Electrical Stimulation Improves Hand Dexterity in Chronic Hemiparesis. Stroke. 2016 10 1;47(10):2596–602. doi: 10.1161/STROKEAHA.116.013791 27608819 10.1161/STROKEAHA.116.013791 PMC5039083

[45] Wilson RD, Page SJ, Delahanty M, Knutson JS, Gunzler DD, Sheffler LR, et al Upper-limb recovery after stroke: a randomized controlled trial comparing EMG-triggered, cyclic, and sensory electrical stimulation. Neurorehabilitation and neural repair. 2016 11;30(10):978–87. doi: 10.1177/1545968316650278 27225977 10.1177/1545968316650278 PMC5048487

[46] Chae J, Bethoux F, Bohinc T, Dobos L, Davis T, Friedl A. Neuromuscular stimulation for upper extremity motor and functional recovery in acute hemiplegia. Stroke. 1998 5 1;29(5):975–9. doi: 10.1161/01.STR.29.5.975 9596245 10.1161/01.str.29.5.975

[47] Howlett OA, Lannin NA, Ada L, McKinstry C. Functional electrical stimulation improves activity after stroke: a systematic review with meta-analysis. Arch Phys Med Rehabil. 2015;96(5):934–43. doi: 10.1016/j.apmr.2015.01.013 25634620 10.1016/j.apmr.2015.01.013

