# Supplementary material for: Arm rehabilitation in post stroke subjects: A randomized controlled trial on the efficacy of myoelectrically driven FES applied in a task-oriented approach
Source: PLoS One. 2017 Dec 4;12(12):e0188642. doi: 10.1371/journal.pone.0188642 (PMC5714329; doi:10.1371/journal.pone.0188642)
Supplement: S1 Protocol — (DOCX) [file pone.0188642.s001.docx]

Arm rehabilitation in post stroke subjects: a randomized controlled trial on the efficacy of myoelectrically driven FES applied in a Task-Oriented approach.

Product of the following project: "Development and application of innovative technologies for neuromotor rehabilitation: clinical study of the effectiveness of Functional Electrical Stimulation controlled by electromyographic signal for upper limb treatment in persons with cerebrovascular outcomes of stroke" (RiSES project)

**Participating centres**

Operating units:

- Biomedical Technology Department, IRCCS Santa Maria Nascente, Don Gnocchi Foundation Onlus, Milano (leading unit)
- Rehabilitation Department, IRCCS Santa Maria Nascente, Don Gnocchi Foundation Onlus, Milano (clinical unit)
- Palazzolo Institute, Don Gnocchi Foundation Onlus, Milano (clinical unit)
- Rehabilitation Department, Don Gnocchi Foundation Onlus, Rovato (clinical unit)
- Rehabilitation Department, Don Gnocchi Foundation Onlus, Roma (clinical unit)
- Neuroscience Department, IRCCS Istituto di Ricerche Farmacologiche Mario Negri, Milano (statistical unit)

**Financing**

The project was financed by the Lombardia Region, Italy (bando ricerca Indipendente 2009)

Rational

Motor recovery of post-stroke patients is favored by synergism between voluntary activity and activity induced by functional rehabilitation exercises. The MeCFES device allows the activation of paretic muscles and/or Paraplegics via electrical stimulation controlled from the EMG signal recorded on the pertinent muscles under voluntary control and can therefore be used as a rehabilitation tool. The first MeCFES device was a single canal unit and the feasibility of its use was verified and the results published in a pilot study: "Myoelectrically driven functional electrical stimulation may increase motor recovery of upper limb in poststroke subjects: A randomized controlled pilot study". Adding more stimulation channels would allow an increase in the range of the device action, making it possible to activate more muscle / body areas (wrist, shoulder) and would thus expand the population of hemiplegic patients, who could benefit from its use.

The present study therefore intends to investigate the effectiveness of multi-channel functional electrical stimulation signal controlled by electromyography in upper limb rehabilitation treatment in patients with cerebrovascular stroke outcomes.

Aims

• Develop multi-channel devices for functional electrical stimulation in electromyographic (MeCFES)

• Include persons with hemiparesis following stroke, a randomized, single-blind trial with a control group was set up to compare a traditional task-oriented rehabilitation treatment to that of using the MeCFES multichannel device in addition to that same treatment.

Summary of the inclusion criteria

- Age greater than 18 years

- Hemiplegia caused by cerebrovascular damage (first ischemic or hemorrhagic stroke) occurring in a period between 1 month and 6 months prior to recruitment (sub-acute patients) and> 6 months (chronic patients)

- Ability and willingness to cooperate during the study

- Reduced function of the upper limb; presence of muscle recruitment of the flexor of the shoulder between 1 and 3 (in chronic patients between 1 and 4) (the MRC scale).

- Detectable muscle activitity in the affected muscles (anterior deltoid), EMG signal detectable by surface electromyography.

- Ability to sit for the duration of the treatment (Sitting Balance Scale> = 2)

Summary of the exclusion criteria

- Presence of epileptic crises (epilepsy)

- Presence of implanted electronic devices (cardiac pacemakers, infusion pumps) or implanted joints in the upper limb

- Presence of serious metabolic diseases and/or of cutaneous ulcers in the affected area by stimulation

- Limb peripheral nerve injury concerning the upper extremity or shoulder muscles totally flaccid.

- Pre-existing disorders that limit the upper limb function

- Passive ROM of shoulder flexion less than 90° limiting the elbow joint

- Overtone any upper limb muscle> = 3 to the Ashworth scale

- Alleged hypersensitivity to electrical stimulation

- Severe cognitive problems, severe apraxia and / or severe aphasia that compromise the understanding of the protocol (MMSE <20)

- Dissociative syndrome or behavioral problems, evaluated according to clinical judgment

- Pregnancy or risk of pregnancy

- Participation in other experiments aimed at the recovery of upper limb function

Number of subjects considered

120 patients with stroke outcomes (80 sub-acute, 40 chronic) to be divided between treated and controls.

Evaluations (for timing, see flow chart attached)

• upper limb function tests (ARAT - Action Research Arm Test)

• Disabilities to upper limbs (questionnaire Quick Dash-9, Fugl-Meyer sect. Upper limbs)

• Disabilities perceived by the patient (IPPA)

• perceived pain (VAS)

• active ROM

• Quality of life (SF-12)

• Shoulder Ultrasound (only in case of subluxation)

Rehabilitation treatment

The upper limb will be treated, both proximally and distally. In the experimental group task-oriented activities will be carried out with the aid of the MeCFES device. Functional exercises of reaching and grasping, for which it is essential to involve the muscles typically deficient in patients with hemiplegia: flexors and abductors of the shoulder, the elbow extensors and the wrist and finger extensors. The frequency of treatments will be 5 daily sessions per week, for 5 weeks, for a total of 25 sessions. The control group will play the same number of rehabilitation sessions practicing task-oriented activities, not including electrostimulation MeCFES.
